# Faculty perspectives on artificial intelligence’s adoption in the health sciences education: a multicentre survey

**DOI:** 10.3389/fmed.2025.1663741

**Published:** 2025-09-29

**Authors:** Eidan M. Al Zahrani, Salah H. Elsafi, Lenah D. Al Musallam, Abdulwahab H. Alharbi, Hala M. Aldossari, Ahmed M. Alomar, Zeyad S. Alkharraz

**Affiliations:** ^1^Physical Therapy Department, Prince Sultan Military College of Health Sciences, Dhahran, Saudi Arabia; ^2^Clinical Laboratory Sciences Department, Prince Sultan Military College of Health Sciences, Dhahran, Saudi Arabia; ^3^Basic Medical Sciences Unit, Prince Sultan Military College of Health Sciences, Dhahran, Saudi Arabia; ^4^Computer Skills Unit, Prince Sultan Military College of Health Sciences, Dhahran, Saudi Arabia; ^5^Department of Clinical Laboratory Sciences, Imam Abdulrahman Bin Faisal University, Dammam, Saudi Arabia

**Keywords:** artificial intelligence, faculty knowledge, faculty perspectives, health sciences colleges, medical education

## Abstract

**Introduction:**

The integration of artificial intelligence technology into healthcare education has been hampered by a number of difficulties. This study aimed to investigate the faculty’s familiarity and perception towards AI applications in medical education concerning various demographic and professional characteristics.

**Methods:**

This observational study used a validated questionnaire distributed to health sciences colleges’ faculty in Saudi Arabia from January 1 to April 30, 2025.

**Results:**

Knowledge rates among the 293 participating faculty were moderate across all groups with an overall average of 58.9%. Knowledge varied significantly with increasing years of experience and academic qualification (*p* = 0.000). In general, positive perception rates were generally high across all groups, with an overall average of 74.8%. Only 33.7% reported agreement on the reliability and accuracy of AI-generated outputs. A large proportion of the participants (72.3%) reported disagreement that AI use poses ethical concerns in medical education. Faculty opinions on the impact of AI on academic integrity varied.

**Discussion:**

This study shows that there are still significant gaps in general knowledge, formal training, and ethical understanding, despite the supportive perception score of the health science faculty towards AI integration in medical education. Despite a general lack of knowledge and a lack of curricular content on AI applications, the results show considerable support for incorporating AI into medical education. Institutional readiness for the integration of AI in medical education is strongly influenced by obstacles, including a lack of knowledge, a shortage of skilled faculty, and fear of career threat.

## Introduction

The general term artificial intelligence (AI) describes the technology that makes it possible for computers and robots to simulate human intelligence (1). The integration of AI technology into health professions education and teaching is anticipated to be a crucial component of any modern curriculum (2). AI has been applied to medical teaching and learning, assessment, medical research, and administration (3). The integration of AI into the teaching of the health sciences is a vital field of research and development. Because AI may enhance learning in both theoretical and practical domains, its role in health professions education is increasingly being recognized. AI techniques are used in simulation-based learning, diagnostic tools, evaluation processes, and healthcare decision-making. AI tools, including data analytics, machine learning, and natural language processing, have the potential to improve student performance and teaching strategies (4, 5). For example, ChatGPT has been reported to enhance higher-order thinking skills, affective-motivational states, and academic achievement, without discernible impact on self-efficacy and mental effort (6).

A rising number of medical specialties, including radiology, ophthalmology, pathology, and dermatology, are utilizing AI (7). Although the majority of AI approaches are used in training laboratories, they are also used in various areas of medical education. By improving future healthcare providers’ knowledge and abilities, AI in medical education has the potential to improve the overall medical care (8). As AI becomes more widely used in the healthcare sector, it is imperative that medical education embraces this technology to guarantee that future healthcare workers have the skills they need to deliver high-quality treatment. However, AI has not been widely used in curriculum review and assessment (9).

Faculty members are essential to the adoption and integration of AI into teaching methods since they have a high influence on the curriculum and pedagogy of health education (10). Faculty perspectives regarding AI in education are influenced by a variety of factors, including technological familiarity, the perceived advantages of AI, and ethical considerations. One of the main issues mentioned in the literature is the differences in faculty members’ levels of AI expertise (11). The lack of familiarity with AI technology may make many health sciences education faculty members reluctant to incorporate it into their syllabi. It has been widely reported that faculty members expressed a need for materials and professional development programs that emphasize AI literacy to integrate AI tools into their teaching strategies (12).

Opinions among faculty members regarding the integration of AI into health professions education vary. Many people see the potential benefits of AI, like increased productivity and improved clinical training, yet some are not comfortable with it. A previous survey found that most faculty members agree AI will play a significant role in health professions education in the years to come (13).

AI adoption in healthcare education has been hampered by a number of problems, including institutional difficulties, faculty reluctance, and technical constraints (4). Faculty members commonly cited the complexity of AI systems, the lack of technical support, and the potential for job displacement as reasons for not integrating AI into their syllabi (14). There has been another concern raised by the faculty members about the ethical issues surrounding AI in education, such as the possibility of data bias, academic integrity, and the need to preserve privacy (10).

Most of the previous studies that assessed the knowledge and perception towards the use of AI in medical education included only the students (15). Very few studies investigated the knowledge and perception of the faculty of the healthcare colleges towards the use of AI in medical education. With the paucity of existing research, increasing need for AI training of healthcare providers, and emerging AI literacy among faculty members, our objectives were to assess the health sciences college’s faculty knowledge and perception toward the use of AI in medical education.

This work aims to examine faculty’s familiarity with, usage of, and perception towards AI in education for different faculty populations based on gender, years of experience as faculty, highest educational qualification, current position, and college. The study questions were: (1). What is the current overall prevalence and pattern of knowledge and perception rates towards AI usage in medical education among the faculty of the health sciences colleges? (2). How do gender, years of experience as faculty, highest educational qualification, current position, and college influence the knowledge and perception towards AI usage in medical education among the faculty of the health sciences colleges, and (3) What is the relationship between faculty’s reported knowledge on their perception towards AI usage in medical education?

## Methods

This observational cross-sectional study used a structured questionnaire prepared by the research group based on the study’s objectives. Ethical approval of this study was obtained from the Institutional Review Board of Prince Sultan Military College of Health Sciences (IRB-2025-CD-011).

The participants were informed about the study’s goals and guaranteeing the privacy of their personal information as a condition of participating in it. All participants signed a written informed consent form electronically.

The research team developed a well-structured, validated, and pretested questionnaire by the study’s goals following a comprehensive review of the literature. The first section included demographical and professional information (age, gender, college, years of experience as a medical educator, highest educational qualification, and current position). The second section included an assessment of knowledge and understanding of AI, including participants’ evaluation of their knowledge and past AI experience. The third section measured the participants’ perception of AI on five-point Likert scale questions. This section measured the participants’ perception of the reliability and accuracy of AI, its ethical concerns, interference with academic integrity, and potential. The fourth part included questions that measured the participants’ practice and challenges of AI in medical education. A convenient sample of 60 faculty from the target group, who were not involved in the study, were given the final version of the questionnaire to test its reliability. The survey questions’ overall Cronbach’s alpha score was 0.87.

A simple random sampling method of healthcare professions faculty of nine colleges in Saudi Arabia from January 1 to April 30, 2025, was included in this study. The questionnaire was distributed to participants through a web link via several social media platforms. Data collectors located at the various college campuses assisted in the distribution of the questionnaire and ensured the inclusion of the targeted participants. The survey was available online through Google Forms from January 1st to March 30th, 2025.

### Statistical analysis

Participants’ knowledge and perceptions about the application of AI in medical education were evaluated using a series of closed and open-ended questions. Only completed responses to all questions were included in the analysis. One point was allocated for the correct response to the multiple-choice questions and zero marks for the wrong one. Responses to the five-point Likert scale questions were rated as: strongly agree (5), agree (4), neutral (3), disagree (2), and strongly disagree (1). The average knowledge and perception scores were calculated out of the total marks of 21 and 25 for knowledge and perception, respectively. The data were processed with SPSS software version 28.0 (SPSS, Chicago, Illinois). The internal consistency reliability of the questionnaire was evaluated using Cronbach’s alpha, and coefficients of 0.7 indicated internal consistency reliability. We used one-way ANOVA to test the significant differences in the knowledge and perception due to various demographic and professional characteristics. The statistical significance was set at *p* < 0.05 for all analyses.

## Results

Out of 360 respondents, 293 (81.4%) completed the survey and were included in this study. Table 1 shows the demographic and professional characteristics of the health sciences colleges’ faculty members who took part in the study. Female faculty made up 56.0%, while males were 44.0%. Of the participants, 13.0% had a bachelor’s degree, 25.9% of a master’s degree, and the majority of 61.1% were PhD holders. Assistant professors made up the largest group of 38.2%, followed by lecturers of 29.4%. Associate professors and professors made up 17.0% of the total, while teaching assistants and clinical instructors accounted for the remaining 13.0%. Of the total participants, 30.4% had below 5 years of experience, while the rest were fairly distributed according to the years of experience, indicating an equal representation of young faculty and experienced professors. The colleges of applied health sciences represented the highest proportion of faculty (60.1%), followed by nursing (19.8%) and medicine (14.0%), while pharmacy and dental schools had low representations of 5.1 and 1.0%, respectively.

**Table 1:** Demographic and professional characteristics of health sciences faculty and their disposition toward AI in medical education.

Demographic characteristics		*n* (%)	Knowledge				Positive perception			
			Score	%	(95% CI)	*p*-value	Score	%	(95% CI)	*p*-value
Gender	Male	129 (44.0)	12.20	58.1	(11.5–12.8)	0.50	18.27	73.1	(17.8–18.7)	0.063
	Female	164 (56.0)	12.50	59.5	(12.0–13.0)		18.91	75.6	(18.5–19.3)	
Years of experience as faculty	0–5 years	89 (30.4)	12.03	57.3	(11.3–12.8)	0.000	18.66	74.6	(18.1–19.2)	0.040
	6–10 years	65 (22.2)	13.20	62.9	(12.2–14.2)		18.10	72.4	(17.4–18.8)	
	11–15 years	66 (22.5)	12.12	57.7	(11.3–12.9)		19.06	76.2	(18.4–19.7)	
	15 + years	73 (24.9)	12.27	58.4	(11.6–12.9)		18.73	74.9	(18.0–19.4)	
Highest educational qualification	Bachelor’s degree	38 (13.0)	12.76	60.8	(11.4–14.1)	0.35	18.31	73.2	(17.5–19.1)	0.721
	Master’s degree	76 (25.9)	11.62	55.3	(10.8–12.4)		18.74	75.0	(18.1–19.4)	
	Doctoral degree	179 (61.1)	12.56	59.8	(12.1–13.0)		18.68	74.7	(18.3–19.1)	
Current position	Demonstrator	22 (7.5)	12.68	60.4	(10.8–14.5)	0.055	18.82	75.3	(17.8–19.8)	0.954
	Clinical Instructor	16 (5.5)	12.87	61.3	(11.0–14.8)		19.12	76.5	(17.4–20.8)	
	Lecturer	86 (29.4)	11.93	56.8	(11.2–12.7)		18.50	74.0	(17.8–19.2)	
	Assistant Professor	112 (38.2)	12.59	60.0	(11.9–13.2)		18.71	74.8	(18.2–19.2)	
	Associate Professor	32 (10.9)	12.37	58.9	(11.3–13.4)		18.66	74.6	(17.8–19.4)	
	Professor	18 (6.1)	12.06	57.4	(10.9–13.3)		17.89	71.6	(17.0–18.8)	
	Other	7 (2.4)	12.71	60.5	(11.1–14.3)		19.57	78.3	(17.7–21.4)	
College	Medicine	44 (15.0)	12.95	61.7	(11.7–14.2)	0.584	18.95	75.8	(18.2–19.7)	0.193
	Pharmacy	15 (5.1)	10.44	49.7	(9.1–11.7)		18.67	74.7	(17.5–19.8)	
	App Health Sci	176 (60.1)	12.45	59.3	(11.9–13.0)		18.68	74.7	(18.3–19.1)	
	Nursing	58 (19.8)	12.29	58.5	(11.6–13.0)		18.45	73.8	(17.8–19.1)	
	Overall		12.37	58.9	(11.9–12.8)		18.65	74.8	(18.3–19.0)	

Table 1 also shows the health sciences faculty’s general knowledge and perception scores towards the use of AI in medical education. In general, knowledge rates were moderate across all groups, with an overall average of 58.9%. Female faculty had a slightly higher knowledge rate (59.5%) than males (58.1%), with no significant difference (*p* = 0.50). Faculty of 6–10 years of experience were the most knowledgeable (62.9%), while the least were among 0–5 years (57.3%), (*p* = 0.000). Knowledge varied with the participant’s academic qualification from 55.3% for master’s degree holders to 60.8% for bachelor’s ones, (*p* = 0.35). Knowledge also varied across academic rankings, with clinical instructors and demonstrators showing slightly higher rates of 61.3 and 60.4%, respectively (*p* = 0.055). Knowledge by college affiliation ranged from 49.7% for Pharmacy to 61.7% for Medicine faculty (*p* = 0.584).

In general, perception rates were high across all groups with an overall average of 74.8%. Females also demonstrated a higher positive perception (75.6%) than males (73.1%) (*p* = 0.063). Perception also differed significantly with years of experience, accounting for 76.2% among those with 11–15 years of experience (*p* = 0.040). All groups of academic rankings had similar perception rates (73.2–75.0%).

Table 2 shows the faculty’s knowledge on specific topics such as previous formal training, AI tools awareness, ethical understanding, curriculum inclusion, and source of knowledge. Out of the total participants, 212 (72.4%) had no formal training or professional development in AI, whereas only 81 (27.5%) reported that they had. Only 30 (10.2) were very familiar with the regularly used AI tools in medical education, while 155 (52.9%) were somewhat familiar, 39 (13.3%) were unfamiliar, and 60 (20.5%) were somewhat familiar. As of participants’ knowledge on the ethical implications of AI in medical education, 7.5% reported no knowledge at all, 28.0% below average, 29.7% average, and 25.6% reported good knowledge. Only 9.2% of them reported excellent knowledge of the ethical implications of AI in medical education. Of the participants, 165 (56.3%) reported having no AI-related course material at their colleges, 51 (17.3%) said it was rarely taught, 63 (21.5%) said it was occasionally taught, and 14 (4.8%) said it was frequently taught.

**Table 2:** Faculty knowledge and experience on AI in medical education.

Variables	Participant’s response	*n* (%)
Previous formal training or professional development in AI	Yes	81 (27.6)
	No	212 (72.4)
Knowledge of AI tools commonly used in medical education	not at all familiar	9 (3.1)
	Slightly familiar	39 (13.3)
	Somewhat familiar	60 (20.5)
	Moderately familiar	155 (52.9)
	Extremely familiar	30 (10.2)
Understanding of the ethical implications of AI in medical education	None	22 (7.5)
	Below average	82 (28.0)
	Average	87 (29.7)
	Good	75 (25.6)
	Excellent	27 (9.2)
Contents of AI in your curriculum	Never	165 (56.3)
	Rarely	51 (17.4)
	Sometimes	63 (21.5)
	Often	14 (4.8)
Sources of previous knowledge on AI	Formal course	37 (9,2)
	Media/social media	140 (35.0)
	Training/workshops	42 (10.5)
	Online training	25 (6.3)
	No training	71 (17.8)
	As part of my degree study	85 (21.2)

Table 3 shows the faculty perspective on AI’s reliability, ethics, integrity, integration, and career impact in medical education. Only 33.7% reported agreement on the reliability and accuracy of AI-generated outputs, and 11.3% reported disagreement, while the majority of respondents (54.9%) remained neutral. A large proportion of the participants (72.3%) reported disagreement that AI use poses ethical concerns in medical education.

**Table 3:** Faculty perceptions of AI in medical education: across reliability, ethics, integrity, integration, and career impact in numbers and percentages (in parentheses).

Variables	Strongly agree	Agree	Undecided	Disagree	Strongly disagree
AI-generated outputs are reliable and accurate.	6 (2.0)	93 (31.7)	161 (54.9)	31 (10.6)	2 (0.7)
Ethical challenges of AI can be effectively managed.	2 (0.7)	13 (4.4)	66 (22.5)	143 (48.8)	69 (23.5)
AI supports academic integrity.	6 (2.0)	56 (19.1)	105 (35.8)	102 (34.8)	24 (8.2)
AI should be included in medical education.	100 (34.1)	151(51.5)	32 (10.9)	7 (2.4)	3 (1.0)
AI enhances rather than threatens career prospects.	47 (16.0)	127 (43.3)	74 (25.3)	34 (11.6)	11 (3.8)

Faculty opinions on the impact of AI on academic integrity varied. While 35.8% were neutral, 21.1% agreed on the interference of AI with integrity, and 34.8% disagreed.

A majority of 85.6% reported agreement on the inclusion of AI in medical education.

While 59.3% perceived some level of threat, 15.4% disagreed, and a quarter remained neutral.

Table 4 shows the faculty perspectives on the relevance, benefits, and challenges of artificial intelligence integration in health professions education. AI was reported as most useful in curriculum development and design (17.3%) and medical research and data analysis (17.2%). Additional applications included administrative communication (15.8%), simulation and virtual patients (14.7%), and personalized/adaptive learning systems (13.8%). Other limitations reported included data privacy and security (20.0%), over-reliance on technology (20.5%), and the accuracy and dependability of AI technologies (21.2%) were the top worries raised. Ethical concerns reported included bias and fairness (15.0%) and the decline of human interaction in education (15.1%). Accessibility and expense concerns were less common (6.7%).

**Table 4:** Faculty perspectives on the relevance, benefits, and challenges of artificial intelligence integration in health professions education.

Variables	Sub-variables	*n* (%)
Perceived useful areas of AI in medical education	Curriculum development and design	214 (17.3)
	Medical research and data analysis	213 (17.2)
	Administrative matters, email, and communication	196 (15.8)
	Simulation and virtual patients	182 (14.70)
	Personalized learning and adaptive learning systems	171 (13.8)
	Assessment and evaluation of students	139 (11.2)
	Others	123 (9.9)
Concerns about AI in medical education	Reliability and accuracy of AI tools	201 (21.2)
	Over-reliance on technology	195 (20.5)
	Data privacy and security	190 (20.0)
	Ethical implications (e.g., bias, fairness)	156 (16.5)
	Lack of human touch in education	143 (15.1)
	Cost and accessibility	64 (6.7)
Priority AI topics in medical education	Disease prediction models	180 (26.2)
	Diagnostics and clinical decision support	140 (20.4)
	Radiology and digital imaging	136 (19.8)
	Medical genetics and genomics	119 (17.3)
	Clinical trials (subject recruitment, tracking)	91 (13.2)
	Natural Language Processing	21 (3.1)
Challenges to AI implementation	Lack of trained personnel to implement AI tools	191 (22.4)
	Limited knowledge or understanding of AI	178 (20.9)
	Ethical concerns (e.g., privacy, bias)	159 (18.7)
	Lack of resources (financial, technological, etc.)	135 (15.9)
	Insufficient evidence of AI’s effectiveness	123 (14.5)
	Resistance to change among educators	65 (7.6)

## Discussion

This study offers valuable insights into the knowledge, perceptions, and readiness of the faculty members of health sciences colleges towards the use of rapidly emerging AI in medical education.

Lack of faculty knowledge and experience, as well as a lack of guidelines regarding AI in medical education, are some of the reasons why many health sciences programs do not currently incorporate AI into their curricula (16). According to the current study, only 27.5% of faculty members had formal AI training, and just 10.2% well familiar with the common AI tools. A similar previous study reported that only 12.04% of the health sciences college’s faculty were very familiar with the AI application in medical education (17). Another study indicated that 50% of faculty in medical colleges who were aware of AI topics were more likely to report that they did not have a basic understanding of AI technologies (18). The current findings have indicated a significant knowledge gap among the faculty, which may be very alarming, even though multiple studies have demonstrated that faculty readiness is a vital component of incorporating AI into medical education.

The study demonstrated statistically significant differences in knowledge rates based on years of experience and academic ranking, with mid-career faculty having the highest knowledge rates. Similar trends were noted in another report (19). This may be attributed to a cohort effect, whereby these individuals are both early adopters of technological innovation and sufficiently established in academia to pursue ongoing education. Numerous studies evaluated the AI gap in knowledge, but mostly included students and healthcare practitioners rather than health science faculty (20).

Notably, all groups had shown favorable perception scores towards the integration of AI in medical education (overall mean = 74.8%), with those with 11–15 years of experience having the strongest positive perceptions (76.2%, *p* = 0.040). This is consistent with earlier research showing favorable attitudes towards AI in medical education (21). Several studies revealed varied attitudes toward AI in medical education; however, mostly about students (22). Most medical students and doctors acknowledged AI’s potential benefits, such as enhancing clinical judgment, research, and auditing skills, and streamlining administrative processes (20).

The rising agreement in the literature that advocates the AI’s inclusion in medical curricula is being supported by the current findings that 85.6% of participants favored AI’s inclusion in the medical studies curricula (20, 23).

The fact that only 7.5% reported excellent knowledge about the ethical concerns of AI and that 21.1% of faculty members thought AI compromised academic integrity is of concern. However, 72.3% of respondents denied that AI raises ethical issues in medical education, which revealed non-traditional points and the need for more in-depth investigations of digital ethics and AI biases, which are issues that have been raised in the literature (24).

The study’s respondents reported the most important applicable areas were disease prediction and prevention (31.6%), medical imaging and diagnostics (20.7%), and personalized treatment plans (20.4.3%). It has been widely reported that AI revolutionized medical practices by improving diagnostic accuracy (25). AI applications in diagnostic imaging have enabled early detection and improved treatment planning (26).

The incorporation of AI competences into medical curricula has been the subject of multiple studies, which proposed that they be made a required part of medical education in order to prepare future healthcare providers for clinical decision-making based on AI (27, 28).

In this study, AI was reported as most useful in curriculum development and design, medical research and data analysis. Additional applications included administrative communication, simulation and virtual patients, and personalized/adaptive learning systems. Assessment and evaluation, administrative matters, email, and communication were also mentioned.

Previous findings revealed a lack of standardized AI curriculum frameworks and notable global discrepancies in the use of AI in medical education. However, several studies highlighted the usefulness of AI in various areas of medical education, such as plagiarism detection, clinical simulations, and homework support (29, 30). In addition, others mentioned the improvement of self-learning and interdisciplinary teaching (31). Clinical simulations, curriculum development, automated feedback, and plagiarism detection are among applications of AI (32). The most prioritized topics for AI integration in curricula were disease prediction models and diagnostics/clinical decision support, followed by radiology and digital imaging, and medical genetics and genomics.

AI has been applied in medical teaching and learning through various systems such as intelligent tutoring systems (33), virtual patients (34), adaptive learning systems, distance education (35), and adaptive feedback (23). AI has also been applied in assessment processes, including optical mark recognition, automated essay scoring, and virtual reality and simulation assessment (35). Other applications of AI in medical education have been through academic medical researchers and administrative matters such as recruitment and admissions, curriculum design and review, management of staff and student records (19).

Although the majority of participants disagreed with the notion that AI creates ethical concerns, their replies on other open-ended questions show that they perceive specific ethical concerns. The most common limitations raised were over-reliance on technology, data privacy and security, and the accuracy and dependability of AI technologies. Additionally, respondents were concerned about ethical issues, including bias and fairness, and the loss of personal touch in education. The ethical implications of integrating AI into medical education are critical to ensuring that technological advancements enhance learning without compromising the core humanistic values of healthcare practice. The Challenges of the Implementation of AI in medical education were previously identified as the difficulty in assessing the effectiveness of the AI application, privacy and confidentiality of the data, and ethical judgment (19).

The most commonly reported challenges by the participants were the lack of trained personnel and limited knowledge or understanding of AI. Other limitations included ethical concerns, lack of resources, and insufficient evidence of AI effectiveness. Resistance to change among educators (7.6%) was the least reported.

The major concerns on the use of AI in medical education widely reported included the reliability and accuracy of AI-generated outputs, academic integrity and moral norms, data privacy and security, fairness and equal treatment, loss of human touch in education, and ethical implications such as bias and fairness (36).

The majority of the previous studies have been conducted on the students’ knowledge and perception towards the application of AI in medical education. However, this study fills a vital and existing gap in the literature about the incorporation of AI in medical education, especially from the viewpoint of the faculty that has not been well studied. The study also highlights previously unreported discipline-specific trends in AI familiarity and perception by adding academics from multiple health sciences fields, allowing for cross-college comparisons. Our limitations are typical of the other studies using a cross-sectional design and self-reported data.

The absence of robustness checks like sensitivity analyses or bootstrapping is one of the study’s limitations. These techniques could help resolve possible breaches of statistical assumptions and offer more assurance regarding the stability of the findings. Such methods could be useful in future research to improve the methodological rigor.

## Conclusion

This study demonstrates that there are still considerable gaps in general knowledge, formal training, and ethical awareness, despite the health science faculty’s supportive attitude toward AI integration in medical education. These results are consistent with worldwide patterns and support pressing demands for faculty development, interdisciplinary curricular reform, and the integration of digital ethics. To fully utilize AI’s potential in influencing medical education in the future, these obstacles must be removed. The study offered data from stakeholder perspectives, primarily to create a foundation for more informed debates and decision-making.

Despite a general lack of knowledge and a lack of curricular content on AI applications, the results show considerable support for incorporating AI into medical education.

In general, institutional readiness for the integration of AI in medical education is strongly influenced by obstacles, including a lack of knowledge, a shortage of skilled faculty, and fear of career threat. The study also raised concerns about the need for proper training as well as the ethical ramifications of over-relying on AI systems.

## Data Availability

The raw data supporting the conclusions of this article will be made available by the authors, without undue reservation.
